# Distality of Attentional Focus and Its Role in Postural Balance Control

**DOI:** 10.3389/fpsyg.2020.00125

**Published:** 2020-02-21

**Authors:** Christian Kupper, Karen Roemer, Elizabeth Jusko, Karen Zentgraf

**Affiliations:** ^1^Institute of Sport Sciences, Department of Movement Science and Training in Sports, Faculty of Psychology and Sports Sciences, Goethe University Frankfurt, Frankfurt, Germany; ^2^Department of Health Sciences, College of Education and Professional Studies, Central Washington University, Ellensburg, WA, United States

**Keywords:** attentional focus, balance, distality, postural control, multiscale entropy, sway analysis

## Abstract

The role of attentional focusing in motor tasks has been highlighted frequently. The “internal–external” dimension has emerged, but also the spatial distance between body and attended location. In two experiments, an extended attentional focus paradigm was introduced to investigate distality effects of attentional foci on balance performance. First, the distality of the coordinates of the point of focus was varied between a proximal and distal position on an artificial tool attached to the body. Second, the distance of the displayed effect on the wall was varied between a 2.5 and 5 m condition. Subjects were instructed to focus on controlling either a proximal or distal spot on a tool attached to their head, represented by two laser pointers. Subsequently, they needed to visually track their own body-movement effect of one of the laser pointers at a wall while completing various single leg stance tasks. Center of pressure (COP) sway was analyzed using a linear method (classic sway variables) as well as a non-linear method (multiscale entropy). In addition, laser trajectories were videotaped and served as additional performance outcome measure. Experiment 1 revealed differences in balance performance under proximal compared to distal attentional focus conditions. Moreover, experiment 2 yielded differences in balance-related sway measures and laser data between the 2.5 and 5 m condition of the visually observable movement effect. In conclusion, varying the distality of the point of focus between proximal and distal impacted balance performance. However, this effect was not consistent across all balance tasks. Relevantly, the distality of the movement effect shows a significant effect on balance plus laser performance with advantages in more distal conditions. This research emphasizes the importance of the spatial distality of movement effects for human behavior.

## Introduction

The importance of postural control mechanisms for the anticipation and execution of bodily movements to avoid postural instability is a well-researched topic (see Cook and Woollacott, 1995, for an overview). Postural control emerges from an interaction between an individual, a specific task with its inherent demands, and environmental constraints (Newell, 1986). On the basis of this situation-specific interaction, postural control mechanisms rely on perceptual and action-oriented information that change with each task (Shumway-Cook and McCollum, 1990; Horak and Macpherson, 1996). Action-oriented processes including musculoskeletal components such as joint range of motion or force production are connected with sensory processes including visual, vestibular, and somatosensory systems creating a frame of reference for postural control (Gurfinkel and Levick, 1991; Hirschfeld, 1992). Visual input provides the individual with a reference of verticality in terms of the position and motion of the body in relation to surrounding objects. Vestibular input on the other hand yields information about the position and movement of the head regarding to gravity and inertial forces. Furthermore, higher level integrative cognitive processes build the foundation of adaptive and anticipatory postural control by mapping sensation to action. One cognitive factor influencing postural control is attentional focusing which refers to subjects’ concentration, not visual focus *per se* (Wulf, 2013, 79, for a review).

In these studies, balance performance as measured by deviations from a balanced position or measures of postural sway has been shown to be enhanced when the performer’s attention is directed to minimizing movements of a platform (further away from the body) as compared to those of their feet (the body itself; e.g., Wulf and Lewthwaite, 2009). However, distality of attentional focus can relate to the distance to the point of concentration to the body as well as the distance between the controller (e.g., a bat) and the body. Generally, the term distality is derived from Heider (1926) who was the first introducing the concept of distinguishing distal versus proximal representations of objects in the environment. According to Heider, the system which is responsible for conscious perception relies on distal cues which create one’s environment.

The importance of distal cues for planned behavior was again highlighted by Brunswik (1944) when stating that the cognitive representations of action goals are built on distal parameters. Distal components serve as foundation for planned behavior, even though proximal components, such as efferent motor commands, are critical to eventually create any kind of distal effect. These conceptual foundations were further elaborated by Prinz (1997) when testing common-coding principles. Common-coding theory by Prinz (1997), derived from the ideomotor principle, suggests that actions are planned in terms of their effects (i.e., pre-determined outcome) for compatibility between planning, action, and perception. Furthermore, the principle incorporates actions as complex event chains that include the perceived stimulus, the action itself, as well as their anticipated re-afferent outcome. Prinz argues for perception and action relying on the same shared cognitive representation. Hence, common-coding principles might suggest that focusing externally on movement effects to better compatible with planning and perception. Since the internal body focus conditions might draw attention away from the intended task goals, performance decrements occur. This notion is further specified by Hommel et al. (2001) in the theory of event coding by assuming that perceived and produced events are not only represented as the same shared codes, but rather as bundles of feature codes. Hommel et al. (2001, p. 876) suggest that “*the feature codes that represent a given perception or action event do not refer to proximal, sensory, or muscular regularities, but to distal attributes of the event, such as the perceived location an external movement-contingent feedback light.*” Taking these assumptions into account, it seems efficient in action control to direct attention toward the intended distal effects in order to process action-related stimuli in contrast to internal sensations. Conceptually, intended actions are often associated with their observable distal movement effect and therefore coded in the same functional unit. These associations become stronger and even interdependent the more they are used. For instance, in order to fulfill the need for light, the action of pressing the light switch is highly associated with the effect of perceiving light. Further postulated models suggest that infants and children also inherently learn to associate action outcomes with voluntary movement control mechanisms, which is valid for novices learning a new motor skill, respectively (Hommel, 2009; Hommel and Nattkemper, 2011).

As the importance of distality is theoretically supported, a clear framework of distinguishing different dimensions of distality seems critical. According to Hossner et al. (2006), distality can be differentiated into three dimensions: spatial, temporal, and modal. “Spatial” refers to the location in space, “temporal” distality can be described by antecedent or effect cues which have a causal temporal relationship, and “modal” distality refers to the modality individuals used to attend to the environmental effects or movement characteristics. Hossner and Wenderoth (2007) in Wulf et al., 2007 postulate that “*it might be beneficial to recognize distality in terms of effect vicinity along different dimensions”* (p. 22).

Most of the studies by Wulf incorporating internal and external attentional focus cues refer to the spatial dimension of distality by using a stabilometer platform. An internal attentional focus represents the mental control of body parts during movement execution like muscle tension or joint position. An external focus refers to the mental control of movement effects outside the body, such as keeping a platform stable (Wulf, 2013). According to Wulf, visual information of movement effects is similar to external attentional foci, which seem hamper internal regulation processes. In order to explain this paradigm, Wulf et al. (2001) proposed the constrained-action hypothesis (CAH). The CAH focuses on underlying processes impacting movement efficiency and effectiveness related to external focus conditions. This hypothesis was based on the idea that movements are executed more autonomously using a non-conscious mode. Therefore, Wulf et al. showed movement effectiveness being hampered by focusing internally (exemplary studies: Zachry et al., 2005; Wulf et al., 2010; Lohse et al., 2011). The spatial dimension of distality enumerates the position of mainly external focus cues with respect to the body. Even though several studies provide evidence of superior movement effectiveness during external attentional focus across varying lab-based and sport specific tasks, different task difficulties, different age groups, and various activity levels (overview by: Wulf and Prinz, 2001; Landers et al., 2005; Wulf et al., 2007; Chiviacowsky et al., 2010; Peh et al., 2011; Wulf, 2013), there is still the need of a specified description of this effect. Exemplary, McNevin et al. (2003) investigated whether increasing the distance of external focus end points enhances balance performance. This study was the first quantifying the impact of spatial dimension of external foci. A near-group (markers in front of feet), far-inside group (markers in between feet), a far-outside group (markers at the outer edges of platform), and again one internal group were included. The far-inside and far-outside groups showed superior performance in contrast to the other groups in keeping the platform in a horizontal position. Their results supported the statement by Wulf and Prinz (2001, p. 656) that “*on the one hand, the effect should be remote from the body, but on the other hand, the effect should still be so close to producing body action and can still be associated by the learner.*” As this paradigm was a step forward in spatially locating beneficial external foci, further research in long jump, golfing, darting, and piano playing was conducted (Hossner et al., 2006; Bell and Hardy, 2009; Duke et al., 2011; McKay and Wulf, 2012; Porter et al., 2012).

In these studies, the external attentional focus advantage is mainly described via a more functional or less restricted control mechanism. Thus, it seems to be critical to investigate not only at the outcome level, but to explore the control mechanisms behind it. In the area of postural control, a common method of quantifying balance in quiet stance is using the magnitude of center of pressure (COP) motion which is typically expressed as sway area (Vuillerme and Nafati, 2007). This measurable outcome quantifies the movement of the COP while standing on a force plate around an equilibrium point (Roerdink et al., 2006). Higher sway area and longer sway paths are associated with poor balance performance, representing less ability to minimize COP movements while standing. Several studies have implemented the described methodological approach to show the beneficial effects of an external attentional focusing (Vuillerme and Nafati, 2007; Olivier et al., 2008; Remaud et al., 2013). However, this widely used method does not provide information about the structure of COP adjustments over time. Therefore, spectral analysis has been used to analyze the frequency of postural adjustments and has previously been adopted in attentional focus studies by Wulf (2008). The spectral analysis might be more sensitive to small adjustments within the sway signal than the classic sway analysis. According to Gurfinkel et al. (1995), a higher frequency describes an increase in the number of active degrees of automaticity, which is interpreted as a higher degree of automaticity within the movement (McNevin and Wulf, 2002; Wulf, 2008).

Recently, multiscale entropy (MSEN) methods have been used to determine the amount of complexity of sway variables in a physiological system. MSEN is a multivariate approach derived from non-linear dynamic models in contrast to linear and thus more conservative approaches examining sway parameters. This approach can provide valuable information about underlying mechanisms that are of non-linear nature (Alcaraz and Rieta, 2010). The movement-related term of complexity [and complexity index (CI)] is derived from the field on non-linear-dynamics and chaos theory (Lipsitz and Goldberger, 1992). By definition, complexity is described with the paradox of unstable (dynamic) stability (Lipsitz, 2002). When standing quietly, the human body relies on highly irregular and complex dynamics which represent interacting regulatory processes. These processes help in adapting to any kind of external stress applied to the system and preparing for an immediate answer (Lipsitz, 2002). Currently, health-oriented and clinical settings utilize this method to assist in identifying differences when analyzing movement sequences; for instance, to compare postural control of fallers versus non-fallers during balance tasks (Costa et al., 2007; Gruber et al., 2011; Fino et al., 2016) or those with concussion history or without (Purkayastha et al., 2019). Higher values in complexity are indicative of a system exhibiting more complex dynamics, which translates to more stable balance performance (Busa and van Emmerik, 2016; Azami et al., 2017). Generally, every form of postural control is highly associated with the COP trajectory resulting in sway. Sway can be a result of correcting imbalance and losing balance. However, some amount of sway is necessary and beneficial to stabilize the body during upright single-leg or double-leg stance (Güenther et al., 2012). A standard sway analysis gives information about the amount of sway; the MSEN in contrast provides more insight into control mechanisms of balance. In other words, classic sway variables can be used as a measure of sway performance while MSEN can be considered as a measure of the quality of sway related to task difficulty (Murillo et al., 2012; Busa and van Emmerik, 2016). Hence, MSEN can give insight to the demand of a sway task related to varying attentional focus conditions. Due to the non-linear approach of the MSEN, the sway signal can be evaluated regarding its regularity and predictability or lack thereof, which has been linked to (non-)autonomous control of the task (Manor et al., 2010).

To date, to our knowledge, no study has investigated differences in attentional focus in balance tasks utilizing the MSEN measure which could provide insights for a better understanding on how different focus conditions mediate the amount of autonomous control and impact balance performance. In contrast to the CAH, more proximal attentional focus cues (mental and visual) might not constrain motor behavior more than distal cues but more distal cues allow more reflexive and autonomous control.

The following experiments focus on exploring the impact of different distality dimensions of attentional focus cues in postural control tasks. In order to implement the outlined theoretical aspects of distality of attentional focus cues, in experiment 1, subjects were instructed to control an external tool on their head with laser pointers attached to it and to simultaneously focus on a more proximal or distal point representing a(n) distal action effect. Notably, both focus points (i.e., the laser pointers) belonged to the same object which was attached to the head. Sensory feedback did not differ between tasks and focus conditions: Subjects could gain visual feedback regarding their performance outcome (i.e., motion of laser light on the wall) throughout every balance trial. More specifically, the proposed research aims at investigating whether there is a difference in balance performance in various SLS tasks while focusing on controlling a more distal versus proximal located point on an external tool attached to the head.

In experiment 2, another layer of distality was added. Specifically, the distance between body and wall was additionally manipulated. More specifically, the proximal and distal attentional focusing already studied in experiment 1 was linked to a proximal versus distal visual anchor representing the movement effect.

## Experiment 1

The objective of the first experiment was to determine the effects of proximal and distal attentional focus cues on two SLS tasks (hop, step). Specifically, the proximal and distal focus points differed in distality from the subject’s body, although both were on the same object attached to the body. It was hypothesized that directed attentional focus would influence postural sway measures. Second, it was further hypothesized that the distal compared to proximal focus would result in a more functional balance strategy during two SLS tasks. In explanation, balance performance under the distal focus condition should result in less sway area and higher CI of the COP movements as found in previous research (Vuillerme and Nafati, 2007; Busa and van Emmerik, 2016; Azami et al., 2017).

## Methods 1

### Participants

This study was approved by the ethics committee of both, the Westphalian-Wilhelms University Muenster, Germany, and Central Washington University, United States. All subjects provided written informed consent prior to participating.

A total number of 22 participants (11 females, 11 males) fulfilling the inclusion criteria aged 18–40 years were included. None of the participants suffered from any injury within the last 6 months and they voluntarily participated in this experiment. The final data analysis included 18 subjects (nine females, nine males; 23 ± 2 years, 174 ± 8.9 cm, 73.7 ± 15.7 kg). Two participants’ data were excluded due to system malfunctions, one subject withdrew from the study and one subject failed to complete the balance tasks properly.

### Apparati and Measurements

For motion analysis purposes, the COP data were collected using force plates supplied by Kistler (Winterthur, Switzerland) (2000 Hz). Additionally, a 12-camera Motion Capture (MOCAP) System (Qualysis, Göteborg, Sweden) with a sampling frequency of 200 Hz was used to acquire 3D trajectories of 52 markers applied to the subject for future kinematic analysis.

**FIGURE 1:**
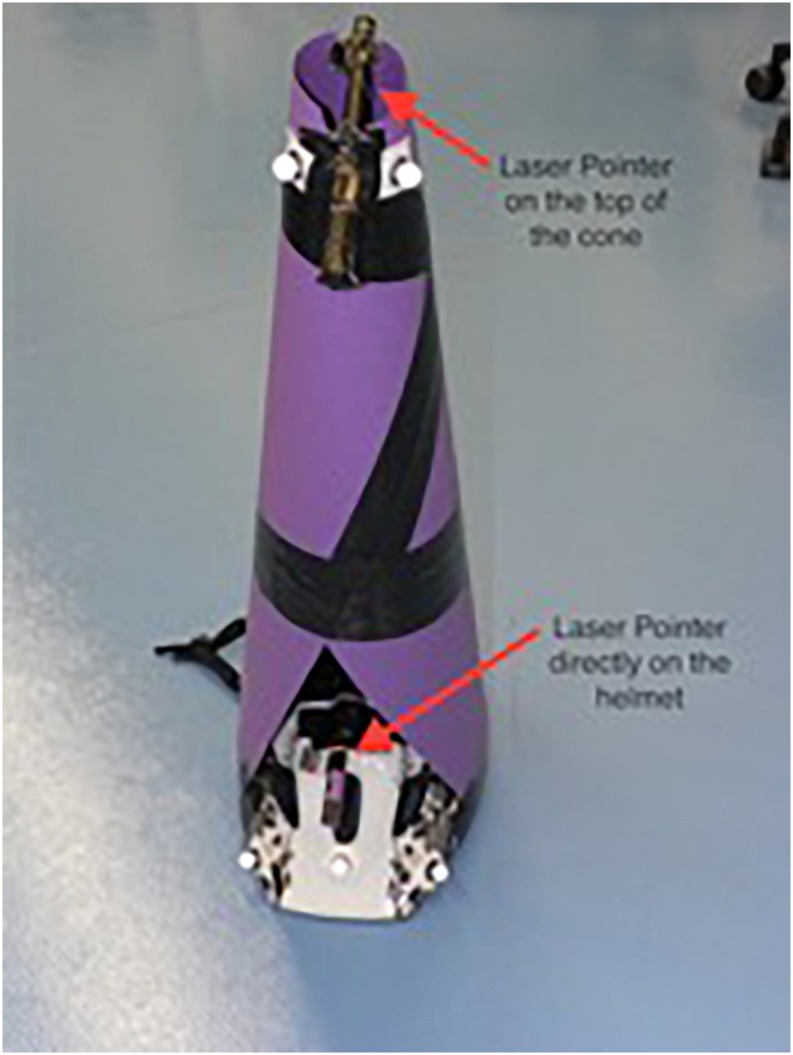
Laser-cone used in Experiment 1.

In order to implement the two different attentional focus conditions, a bicycle helmet with a paper cone attached (laser-cone) was used. Two laser pointers were attached to the paper cone, one on top of the cone and the other one at forehead level to the helmet itself (Figure 1). Both pointers could be adjusted to aim at the exact same spot on a projection wall at a 5-m distance. This led to identical visual feedback for the subjects regardless of which pointer was switched on. For each focus condition, one laser pointer was covered during the measurement. During the proximal focus condition, subjects were only able to observe their lower laser point on the wall and vice versa for the distal focus condition.

The trajectory of the laser pointer on the wall was tracked by a third-generation GoPro Hero action camera (frequency: 29.97 frames per second) which was set up on a tripod behind the wall.

### Study Design and Procedure

The experiment was conducted using a within-subject study design with two focus conditions and two balance tasks (2 × 2). All subjects were repeatedly tested in two randomized balance tasks under the instruction of two attentional focus conditions (proximal and distal). Each task needed to be performed for 10 s.

**FIGURE 2:**
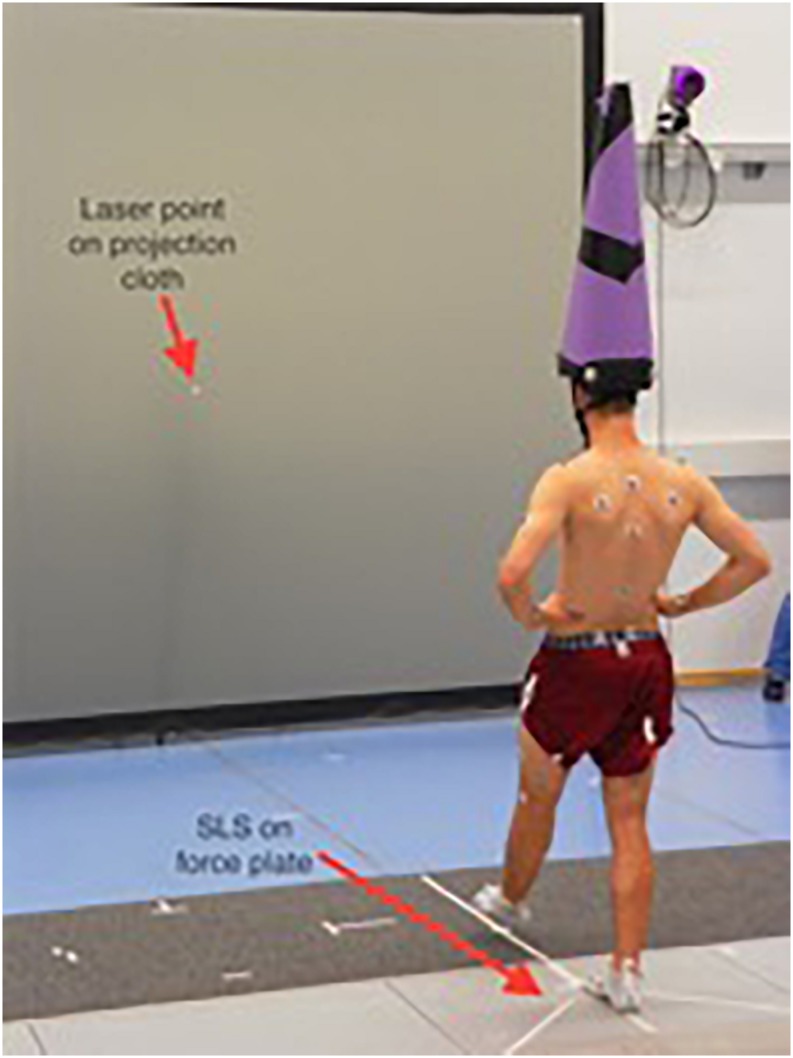
Subject performing single-leg stance (SLS) on force plate.

A previously validated balance recovery test (de Ruiter et al., 2010) was applied to determine leg dominance. The test required participants to stand still in an upright position, while one of the investigators pretended to check their body posture from behind. Without notification, subjects were slightly pushed forward causing them to perform a step to regain balance. The leg used for stepping forward indicated their dominant leg for the single leg stance balance tasks. After three trials, everyone was debriefed. For the measurements, subjects were asked to stand on a force plate facing a wall at 2.5-m distance. For each subject, a small piece of tape was placed on the wall at the participant’s eye level which served as their reference point for the laser dot (Figure 2). Each attentional focus condition contained the same two SLS tasks with the dominant leg as the supporting leg: (a) self-paced step into SLS position (approximately regular step-length) and (b) hopping off a 10 cm mat into SLS position on the force plate (same stepping length). Subjects were asked to maintain quiet stance on their dominant leg after performing step/hop for 10 s for a valid trial. Both tasks were performed with hands akimbo to prevent arm movements and guarantee reliable measurement conditions. Subjects repeated both tasks until three valid trials were recorded under both attentional focus conditions.

### Attentional Focus Instructions

The attentional focus was directed by standardized instructions before every trial. The subjects’ focus was verbally directed by the investigators before the initial trial for each task. During the proximal focus condition, subjects were instructed to: “Focus on controlling the laser pointer directly attached to your forehead.” For the distal focus condition, subjects were introduced to: “Focus on controlling the laser attached to the top of your cone.” The verbal instructions given to the subjects by the researcher emphasized “head” and “cone” to describe the distality of the laser pointer in use.

The distality of the attentional focus for the subsequent trials was reinforced by asking the participants “*which laser pointer are you controlling?.*” Subjects were supposed to answer corresponding to the focus condition either “*the laser pointer attached to my forehead*” (proximal) or “*the laser pointer attached to the top of the cone*” (distal). In addition, the subjects received real-time feedback of their performance by visually tracking the movement outcome represented by moving the laser dot on the wall. A constant repetition of the attentional focus instructions served to ensure the subjects’ awareness of the respective focus condition.

### Sway Analysis

The COP trajectory was captured using a 600 × 900 AMTI force platform. Measurement sampling frequency was 2000 Hz, which was downsampled to 200 Hz for the MSEN to avoid oversampling issues. All sway data were normalized to the subject’s body height. Data analysis started when the load on the force plate exceeded 75% of the body weight. Classic sway area described by the 95%-confidence-interval sway ellipse determined by principal component analysis was calculated (Oliveira et al., 1996).

### Multiscale Entropy (MSEN) Measures

Multiscale entropy allows for the assessment of point-to-point fluctuations over a range of time scales and thus quantifies the degree of regularity or predictability over multiple time scales (Costa et al., 2002; Alcaraz and Rieta, 2010). MSEN has been used to investigate the amount of complexity of sway variables in a physiological system, whereby increases in entropy values represent a greater degree of complexity which is indicative of better balance performance (Azami and Escudero, 2016; Busa and van Emmerik, 2016).

For MSEN to quantify the complexity within a system across times scales, the amount of irregularity at each time point must be determined first (sample entropy) (Eq. 1):

(1) $$S_{E}\,\left(m,r,N\right)=-l\,n\,\left(\frac{A^{m}\,\left(r\right)}{B^{m}\,\left(r\right)}\right)$$

where *S*_*E*_ is the sample entropy value, *m* is the number of consecutive points in one template (*m=2*, Gow et al., 2015), *r* is the radius of similarity (*r* = 0.15, Gow et al., 2015), *N* is the length of the times series, *B^m^*(*r*) represents the probability that two templates will match for *m* points within *r*, and *A^m^*(*r*) represents the probability that two templates will match for points within *m+1* across the whole times series.

**FIGURE 3:**
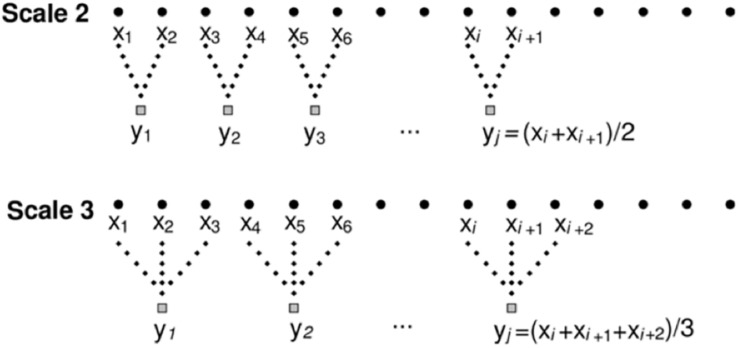
Example of coarse-graining procedure for time scales two and three. Adopted from Costa et al. (2002).

The original times series was coarse-grained for complexity to be assessed over ten timescales with τ = 10 (Figure 3), where *x* is the original time series and *y* is the new time series. Ten timescales across the 10-s data collection volume allow for an increase in the number of points put into one timescale and therefore detection of lower frequencies in the signal. Additionally, the CI can be quantified by taking the area under the sample entropy and time scale curve. It is recommended for postural control measures that results of MSEN analysis is reported as a time scale in seconds or hertz (Hz) rather than a scale factor (number) (Busa and van Emmerik, 2016). Therefore, MSEN results of this study are reported as a time scale in Hz (Eq. 2).

(2) $$T\,i\,m\,e\,S\,c\,a\,l\,e=\frac{S\,a\,m\,p\,l\,e\,F\,r\,e\,q\,u\,e\,n\,c\,y}{\left(m+1\right)\times \tau }$$

### Laser Data

The laser pointer trajectory on the wall was captured by a GoPro (Generation 3). The GoPro was synchronized with the COP data using an acoustic trigger signal generated by the space bar indicating the start and end point of each measurement.

The laser pointer trajectory was tracked using the using the freeware software Tracker (Physlets, Physics, 2016). Furthermore, time on target represented the time the laser pointer stayed with a 2.5 cm radius around a reference point representing the center of the sway area created by the laser pointer on a projection wall in 2.5 m distance (Ledebt et al., 2005). The reference point was calculated via principal component analysis similar to the approach used for the classic sway area of the COP as described above. In a second step, the pythagorean theorem was applied to calculate the distance of the laser pointer at any given point in time from the virtual reference point. Lastly, time on target was determined as the sum of all timeframes with a distance less than 2.5 cm. Time on target served as an additional performance criterion of keeping the laser as close as possible to the target piece of tape on the projection wall.

All variables were calculated in Matlab (MathWorks, 2016) for a timeframe of 10 s after exceeding 75% of body weight on the stance leg.

### Statistical Analysis

All data were tested for normality using Shapiro Wilk’s test. As normality could not be assumed for each instance, a generalized linear mixed model (GLMM) analysis of variance was employed. Task and focus were used as fixed effects with pairwise contrasts and robust covariance settings were used to address heterogeneity in the data. The significance level was set at *p* = 0.05. Laser data of the difference in time on target between proximal and distal were analyzed using Wilcoxon signed-rank test—as the assumption of normality was violated for some tasks.

All statistical analyses were completed using the Statistical Package for the Social Sciences (SPSS) software (International Business Machines Corporation SPSS, release version 22; IBM Inc., Armonk, NY, United States).

## Results 1

For detailed information on the results see Supplementary Material.

### Laser Data

#### Time on Target

Mean values of time on target calculations in the hop task showed 6.37 s (SD = 1.47) under distal and 6.53 s (SD = 1.58) under proximal focus, whereas step task showed 6.73 s (SD = 1.38) under distal and 6.65 s (SD = 1.42) under proximal focus.

Wilcoxon signed-rank test did not report a statistically significant median difference between focus conditions in either of the tasks. Hop task revealed *z* = 0.174, *p* = 0.862 and step task *z* = −0.196, *p* = 0.845, respectively.

### Classic Sway Analysis

#### Sway Area

Generally, sway area is displayed in mm^2^/BH, AP as well as ML sway in mm/BH. The abbreviation BH represents body height. Mean sway area was 589.89 (SD ± 146.08) under proximal focus and 553.49 (SD ± 219.39) in the distal focus condition. Step task showed 456.67 (SD ± 106.15) under proximal focus. In the distal focus condition, mean sway area was 430.39 (SD ± 126.04). No main effect of focus on sway area was found in both tasks (Hop *p* = 0.411, Step *p* = 0.264).

### Multiscale Entropy Measures

#### CI COP Position Radius

The hop task showed 4.49 (SD ± 1.30) under proximal and 4.93 (SD ± 1.33) under distal focus. Step task revealed 5.78 (SD ± 2.04) under proximal and 5.24 (SD ± 1.22) under distal focus. GLMM revealed a main effect of focus on the CI of COP position radius between proximal and distal focus in the hop task, indicating increased complexity under distal focus [*F*(1,170) = 5.107, *p* = 0.025].

For those variables with significantly different complexity indices, sample entropy across timescales was compared to further analyze complexity. No main effect of focus across all timescales were found [*F*(1,340) = 3.539, *p* = 0.061]. However, sample entropy values were significantly higher for distal focus on timescale seven [*F*(1,340) = 3.879, *p* = 0.050], eight [*F*(1,340) = 4.008, *p* = 0.046], and nine [*F*(1,340) = 3.954, *p* = 0.048], which relates to movement frequencies of 5.6–3.7 Hz.

## Discussion 1

This experiment used a novel attentional focus paradigm to investigate biomechanical effects of giving distally versus proximally focused instructions on the performance of two different SLS balance tasks. The analysis focused on attentional-focus-related COP movement analysis via classic sway analysis as well as on multi-scale entropy of COP trajectories (MSEN). Subjects were instructed to focus on an either proximally or distally attached laser pointer while visually focusing on a reference point on individual eye level. The reference point needed to be aimed at with proximal or distal laser pointer, respectively, henceforth providing visual online feedback. Laser data, serving as manipulation check, showed no difference between tasks. Meaning subjects focused the same amount of time on the projected reference point on the wall as previously instructed. Differences in COP movement cannot be explained by laser data and need to be associated with attentional focus instructions.

In line with the hypothesis, attentional focus instructions influenced the COP signal. However, classic sway area and MSEN measures revealed different results. Overall, sway area did not reveal a significant difference in size between focus conditions in either of the tasks. These results are consistent with previous research on balance and attentional focus. Several other studies reported higher COP sway values under an external or more distal attentional focus, respectively. However, as previously suggested by McVey et al. (2009), sway area might not be sensitive enough to detect slight differences in sway behavior.

The concept of associating a smaller sway area with enhanced balance performance needs to be specified. One cannot decrease the sway area unlimitedly as at some point less sway simply is not functional in order to maintain balance. Furthermore, it seems critical to reveal information about how the COP is controlled across certain timescales to get more valid information about the control mechanisms behind postural control. Therefore, this experiment utilized the MSEN CI to further investigate COP sway movement and potentially give an understanding of the control mechanisms which COP movements are based on. Generally, the CI describes the degree of regularity and predictability within the signal; higher values describe a less predictable COP movement. Higher CI values are indicative for more autonomous control of movement in contrast to smaller CI values which describe more conscious involvement in controlling the COP. CI of center of position radius showed significantly higher complexity in postural fluctuations with distal compared to proximal focus in the hop task.

The sample entropy over timescales for the hop task revealed significant differences between focus conditions in the higher timescales, which relates to low movement frequencies. The step task did not reveal a significant difference in the CI for the center of position radius with respect to focus conditions. Both tasks vary in task demand. As the step task is more overlearned, it might be related to less conscious control and a more autonomous mode regardless of focus condition.

Concluding, classic sway area did not reveal a difference between focus conditions for both tasks. However, MSEN showed higher complexity for the distal focus condition in the hop task, whereas step task did not show any difference. As MSEN measure can give insight in the functionality of sway behavior, classic sway area measures might not detect differences at that level.

In order to expand the findings of experiment 1 and to gain a deeper understanding of *how* proximal and distal attentional focus instruction mediate balance performance, a follow-up experiment including the presented paradigm was conceptualized. Experiment 2 included slight changes regarding methodology; however, the overall task comprising laser-cone apparatus plus instructions was maintained. As experiment 1 only investigated the effects of distality of the coordinates on the mentally controlled tool, experiment 2 widens this concept by proposing a multi-dimensional continuum adding a layer of distality of the movement effect (i.e., wall distance on which laser pointer is projected). Focusing on movement effects has been shown to enhance performance (Wulf et al., 2000). Previously, research mixed up investigating distality effects of focusing on the tool controlled and the observable movement effect by comparing these conditions (Bell and Hardy, 2009; McKay and Wulf, 2012). The presented design allows to look at concepts separately, even though they were carried out simultaneously. Namely, proximal and distal focus instruction comprised a 2.5 and 5 m movement effect condition. Subsequently, subjects were able to visually observe their movement effect (i.e., laser pointer movement) in 2.5 and 5 m distance on the wall while controlling either a proximal or distal laser pointer.

Again, the follow-up experiment includes the basic postural control tasks of step and hop. However, both were re-conceptualized to be more dynamic balance tasks in experiment 2 in order to check for the specificity of the focus effects found in experiment 1. In explanation, the step task included three single-leg squats in between the quiet stance period and the hop task was performed more horizontally this time. These changes result in a higher specification of attentional focus effects, as Raab (2007) in Wulf et al. (2007, target article) mentioned the importance of avoiding the postulation of global effects.

From a methodological standpoint, enhanced data processing opportunities were guaranteed by lengthening trial duration to 30 s of balancing. Consequently, MSEN specifications were adjusted to enhance sensitivity and reliability of detecting differences in performance as recommended by Gow et al. (2015).

## Experiment 2

The objective of this experiment was to gain further insight in the difference of proximal and distal attentional focus instructions on balance performance in three SLS tasks (hop and step). Moreover, the influence of distality of the movement effect is researched.

It was hypothesized that directed attentional focus will influence postural sway measures. As more dynamic tasks were included, it was further hypothesized that tasks under distal attentional focus instructions show less sway and higher complexity indices to maintain balance compared to proximal focus. Third, as the distance of movement effect has never been varied before separated from the focus point itself, it was hypothesized to have an influence on balance performance. Proposedly, the effect further away condition (wall 5 m) will induce more complex movements to maintain balance.

## Methods 2

### Participants

The second experiment was approved by the Human Subjects Review Program of Central Washington University, United States, and subjects provided written informed consent prior to participating.

Data were collected on a total number of 18 participants (8 females, 10 males; 25 ± 4 years, 171.25 ± 12.4 cm, 74.4 ± 15 kg) matching the inclusion criteria aged 18–40 years. None of the participants suffered from any injury within the last 6 months. All participants were assessed by the Physical Activity Readiness Questionnaire (PAR-Q) and they voluntarily participated in this study. Final data analysis was run with only 15 subjects, as the laser data of three subjects could not be further processed due to noise in the video.

### Apparati and Measurements

Kinetic data of the different ground reaction force (GRF) components of the COP were collected using an Advanced Mechanical Technology, Inc. (AMTI) force platform (600 × 900 mm) (1200 Hz). Kinematic data of the time histories of 3D coordinates were acquired by an eight camera MOCAP System (Motion Analysis, Santa Barbara, CA, United States) using a sampling frequency of 120 Hz for future analysis.

**FIGURE 4:**
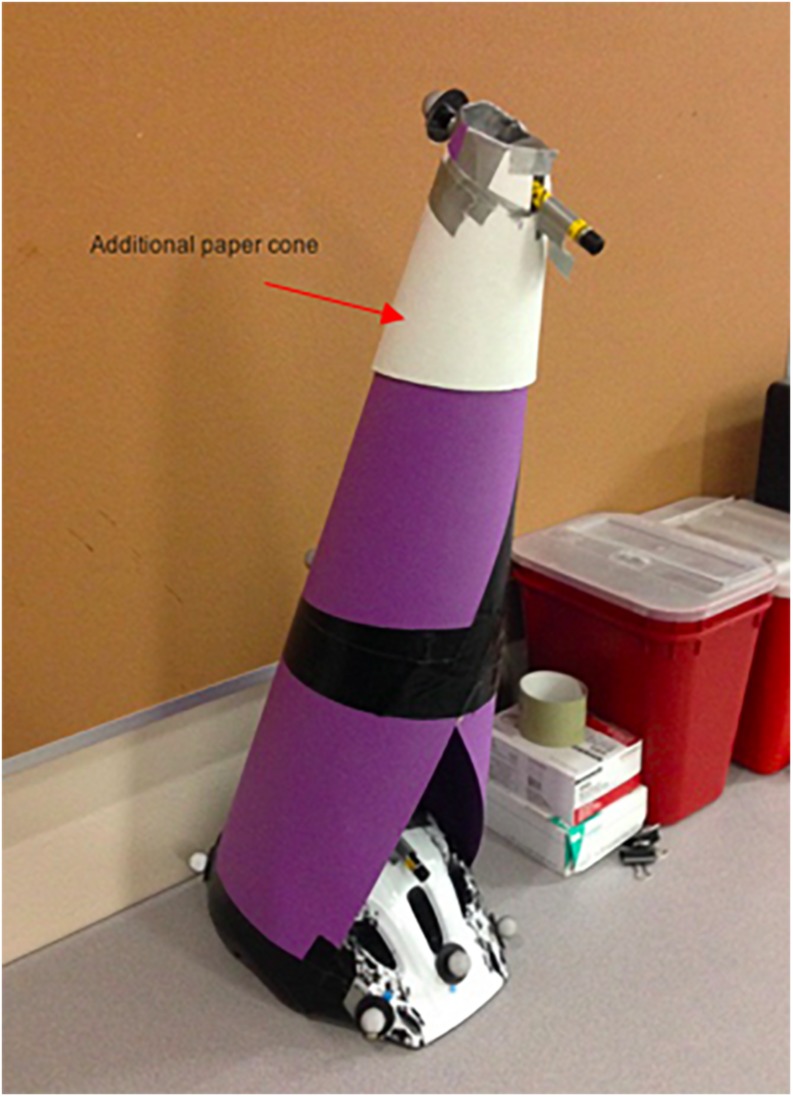
Laser-cone apparatus used in Experiment 2.

Both attentional focus conditions were implemented by using the same helmet and cone apparatus as described in experiment 1. However, for this experiment, a small white paper cone on top of the big purple one was set up in order to reliably adjust the angle of the top laser and ensure that both laser beams intersected on the wall for the 2.5 m and the 5 m distance, respectively (Figure 4).

Laser movements were recorded using the same third-generation GoPro Hero action camera (frame rate: 29.97 Hz) which was set up on a tripod behind the wall. Synchronization of the three measurement devices was guaranteed by an acoustic signal of the computer, which simultaneously started the capturing process of the cameras and force plates and is audible in the GoPro video.

### Study Design and Procedure

**FIGURE 5:**
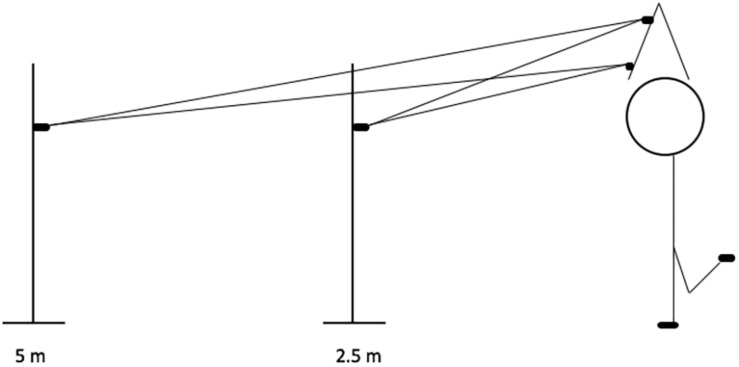
Schematic representation of the task in Experiment 2.

The experiment was conducted using a within subject study design. All subjects were repeatedly tested in two different SLS balance tasks under the instruction of two attentional focus conditions (proximal and distal). A counterbalanced amount of 24 trials of balancing needed to be completed by every participant. Furthermore, the position of the projection wall changed regarding to distance between 5 and 2.5 m (Figure 5). Thus, a 2 × 2 × 3 design with respect to attentional focus (laser position), movement effect (wall position), and number of tasks was conducted.

All participants were tested in two separate meetings. During the first meeting, an average step length calculation via motion capturing and a balance recovery test took place during the first appointment.

Upon arrival, the laser-cone was applied and the subjects performed the same tasks for both wall distances and each attentional focus condition containing (a) self-paced step in single leg stance position including three mini-squats and (b) self-paced hop in single-leg stance position (equals 120% of average step length). Subjects needed to maintain balance in SLS after landing on the force plate for 30 s. Again, all tasks were performed with the dominant leg as the supporting leg and the hands akimbo. After 10 s of the step task, subjects received verbal instructions to squat three times (i.e., bending knees to about 40°) and maintain single leg stance for another 10 s afterward. Due to restrictions in the height of the ceiling, the hop task was performed horizontally. Two pieces of tape on the floor indicated range of movement for both tasks. Subjects performed every task until completing three successful trials under both attentional focus conditions (proximal and distal) and both distances of the wall (2.5 and 5 m).

### Attentional Focus Instructions

Attentional focus instructions did not differ in wording or repetition from those given in experiment 1.

### Sway Analysis

All data were normalized to individual subject body weight and height. Subsequently, data analysis again started at the point the load on the force plate exceeded 75% of subject’s body weight. Again, sway area as included in experiment 1 was calculated.

### Multiscale Entropy Measures

In their review, Gow et al. (2015) identified several methodological key aspects such as data length, sampling rate, or point matching tolerance in order to validly carry out a multiscale entropy analysis. Parameters in this experiment were adjusted accordingly. Through the adaptation of trial length to 30 s and sampling rate to 1200 Hz, results were no longer limited to only ten timescales. Hence, incorporating 20 timescales across a 30 s collection volume allows for an increase in the number of points put into every single timescale, which consequently increased the chances to detect signal predictability at lower frequencies.

### Laser Data

The virtual target size was adjusted according to wall distance. Similar to experiment 1, the radius for the 2.5 m wall distance was set as 2.5 cm. To maintain a constant virtual target size, the intercept theorem was applied to determine the radius for the 5 m distance wall as 5 cm. It was assumed that the overall location of the cervical spine as pivot point for the laser pointer is comparable for both conditions. Apart from varying the radius around the reference point for time on target calculations between 5.0 cm for the 5.0 m distance and 2.5 cm for the 2.5 m distance of the projection wall, laser time on target calculations were processed as in experiment 1.

### Statistical Analysis

All data were tested for normality using the Shapiro Wilk’s test. COP sway, laser sway, and laser time on target were analyzed in the same manner. Mean values out of at least two valid trials per condition and task per subject were further analyzed utilizing a 2 × 2 × 3 ANOVA. Wall distance and focus were set as mains factors. The significance level was set at *p* = 0.05. All statistical analyses were completed using the SPSS software (International Business Machines Corporation SPSS, release version 22; IBM Inc., Armonk, NY, United States).

## Results 2

For detailed information on the results see Supplementary Material.

Generally, all presented results incorporate data of the entire 30-s volume. Main effects of wall and focus refer to differences between wall distances (5 m/W5 and 2.5 m/W2.5) and focus conditions (proximal and distal) across all tasks, respectively. Notably, as mentioned before, COP data calculations included data of 18 subjects, whereas laser data calculations only encompassed 15 subjects. Units in tables are adopted from experiment 1.

### Laser Data

#### Laser Time on Target

**FIGURE 6:**
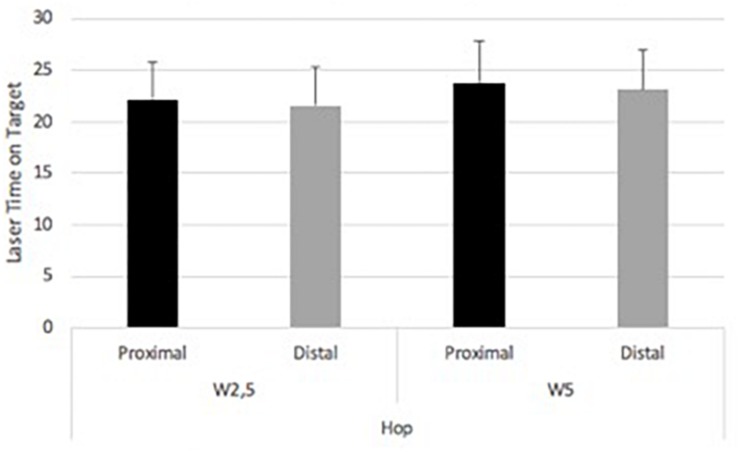
Experiment 2. Laser time on target within foci between wall distances in the hop task.

Hop task showed 21.55 (SD ± 3.9) s for W2.5m and distal laser. For W5m and distal laser time on target was 23.16 (SD ± 3.8) s. W2.5m and proximal laser revealed 22.15 (SD ± 3.6) s. W5m and proximal laser showed 23.82 (SD ± 4.0) s (Figure 6). A main effect of wall was found [*F*(1,14) = 7.14, *p* = 0.018] with an effect size of eta squared: 0.338. Neither a significant main effect of focus nor interaction effect was found.

**FIGURE 7:**
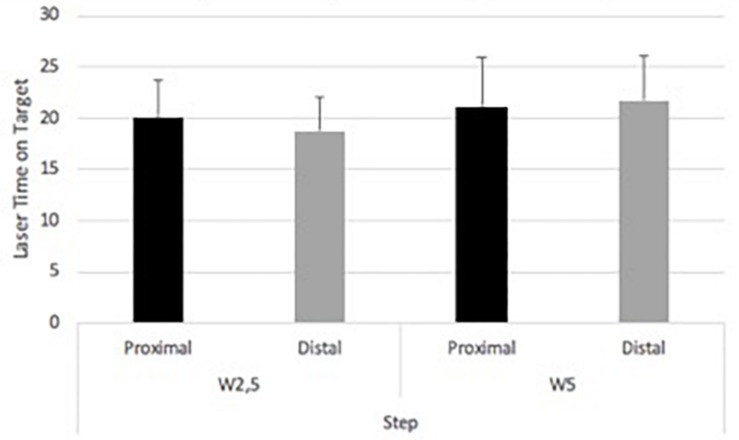
Experiment 2. Laser time on target within foci between wall distances in the step task.

Step task showed 18.74 (SD ± 3.6) s for W2.5m and distal laser. W5 and distal laser time on target was 21.7 (SD ± 4.3) s. W2.5m and proximal laser revealed 20.01 (SD ± 3.7) s (Figure 7). W5m and proximal laser showed 21.1 (SD ± 4.8) s. No significant main effect was found. A main effect of wall [*F*(1,14) = 9.88, *p* = 0.007; eta squared: 0.414] and interaction effect [*F*(1,14) = 6.7, *p* = 0.021; eta squared: 0.324] were found.

#### Laser Sway Area on Wall

Hop task revealed 36.37cm^2^ (SD ± 18.0) for W2.5m and distal focus. W5m and distal focus showed 95.44 cm^2^ (SD ± 49.1). W2.5m and proximal focus showed 29.63 (SD ± 13.7). W5m and proximal focus showed 98.44 cm^2^ (SD ± 61.8). No significant main effect of focus and interaction was found. Main effect of wall was significant [*F*(1,14) = 31.57, *p* < 0.001; eta squared: 0.693].

Step task showed 50.28 cm^2^ (SD ± 27.5) for W2.5m under distal focus. W5m under distal focus showed 118.01 cm^2^ (SD ± 74.6). W2.5m and proximal focus revealed 34.01 cm^2^ (SD ± 12.4). W5m under proximal focus showed 111.07 cm^2^ (SD ± 60.3). No significant main effect of focus and interaction was found. A significant effect of wall was found [*F*(1.14) = 29.71, *p* < 0.001; eta squared: 0.68].

#### Sway Area

Hop task showed 555.90 (SD ± 127.66) for W2.5m under distal focus. W5m under distal focus showed 625.10 (SD ± 176.51). Hop task revealed 603.76 (SD ± 167.02) for W2.5m under proximal focus. Mean sway area for W5m under proximal focus was 563.28 (SD ± 167.21) for. No significant main effect of focus and wall were found. Statistical analysis revealed a significant interaction effect [*F*(1,17) = 6.43, *p* = 0.021; eta squared 0.274].

Step task showed 622.52 (SD ± 139.70) for W2.5m under distal focus. W5m under distal focus showed 649.98 (SD ± 200.33). W2.5m under proximal focus revealed 617.99 (SD ± 134.53). W5m under proximal focus showed 642.78 (SD ± 109.97). Statistical analysis revealed no significant effect overall for step task.

### Multiscale Entropy

Complexity indices for radius showed increased values for wall 5 m compared to wall 2.5 m. Again, statistically significant differences were further analyzed by comparing sample entropy across timescales.

#### CI COP Position Radius

**FIGURE 8:**
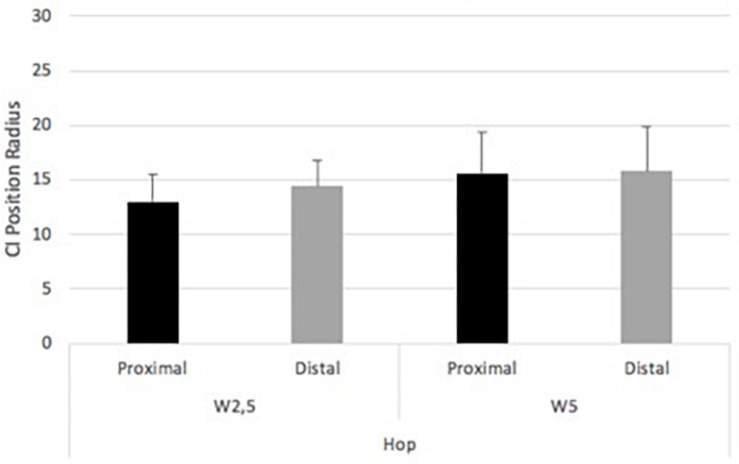
Experiment 2. Complexity Index position radius within foci and between wall distances in the hop task.

Hop task showed 14.41 (SD ± 2.29) for W2.5m under distal focus. W5m and distal focus showed 15.79 (SD ± 4.06). W2.5m and proximal focus revealed 12.98 (SD ± 2.51). W5m and proximal focus showed 15.61 (SD ± 3.70) (Figure 8). A main effect of wall was found in the hop task [*F*(1,17) = 13.65, *p* = 0.002; eta squared: 0.445]. No significant main effect of focus was found in the hop task [*F*(1,17) = 2.82, *p* = 0.11; eta squared: 0.142]. No significant interaction effect was found in the hop task [*F*(1,17) = 2.19, *p* = 0.16; eta squared: 0.11].

**FIGURE 9:**
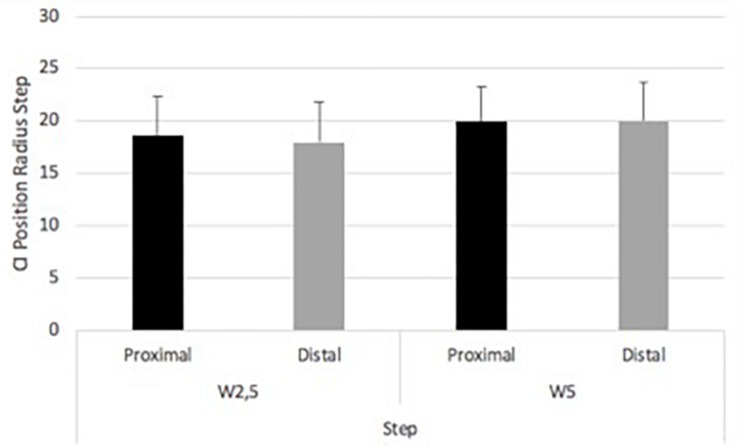
Experiment 2. Complexity Index position radius within foci and between wall distances in the step task.

The step task showed 18.56 (SD ± 3.90) for W2.5m under distal focus. W5m under distal focus showed 19.93 (SD ± 3.32). W2.5m under proximal focus showed 17.95 (SD ± 3.88). W5m under proximal focus showed 20.01 (SD ± 3.64) (Figure 9). A significant main effect of wall was found in the step task [*F*(1,17) = 8.31, *p* = 0.01; eta squared: 0.328]. No significant main effect of focus and interaction were found in the step task, respectively [*F*(1,17) = 5.11, *p* = 0.48; eta squared: 0.03 and *F*(1,17) = 1.52, *p* = 0.24; eta squared:0.08, respectively].

## Discussion 2

This experiment used the same attentional focus paradigm to investigate biomechanical effects of providing distally versus proximally focused instructions on the performance of two different SLS balance tasks. Besides a proximal or distal condition in distality of the mentally controlled tool attached to the subjects body, the distality of the visually presented movement effect was varied between a far and close condition. Again, a COP movement analysis via classic sway analysis as well as MSEN measures was conducted. Subjects were instructed to either focus on a proximal or distal laser pointer attached to their head while visually focusing on a reference point on individual eye level while performing either of two balance tasks (step or hop). The reference point on an external wall needed to be aimed at with proximal or distal laser pointer, respectively. Consequently, visual online feedback of the performed task was provided.

Generally, neither hop nor step task revealed a main effect for attentional focus in COP sway. However, an interaction effect between both attentional focus conditions was found for the hop task. Within the non-linear MSEN measure, a wall effect was found for both tasks with higher complexity in the distal wall condition, suggesting a more autonomous movement control when visually focusing more distally. Linking higher complexity of movement to a more autonomous sway behavior is supported by previous findings (Van Emmerik and Van Wegen, 2000). Within the laser data, a wall effect was found for hop and step task. More specifically, subjects managed to stay longer within their artificially calculated target area during the 5 m wall distance condition. In addition, an interaction effect between both factors can be found in step task. Longer time on target results is supported by main effects of the factor wall distance in hop and step task. In both tasks, sway area increased under wall 5 m condition leading to mentioned wall effect. However, at first glance, contradictory finding can be explained by the mathematical intercept theorem. Based on intercept theorem, a four times higher sway area is expected when doubling the distance of laser projection. In the hop as well as the step task, sway area was increased less than four times suggesting a relatively decreased sway area. Henceforth, laser data act supportive to the more autonomous COP control under distal wall distance condition by showing enhanced performance under the same condition.

## General Discussion

The aim of the present studies was to assess the role of two different distality dimensions of attentional focus in balance tasks. Distality in terms of the spatial coordinates was addressed by directing the attention toward a proximal or distal point on an artificial tool attached to the body. Subjects were instructed to focus on either the proximal or distal point and to visually track their produced movement effect while executing various single leg stance tasks. Moreover, the approach was extended throughout experiment 2 by investigating differences in performance under two spatial distances between the visually displayed movement effect and the body.

In general, existing literature for attentional focus has provided evidence for performance differences between internal versus external attentional focus (e.g., Vuillerme and Nafati, 2007; Olivier et al., 2008; Remaud et al., 2013). The experiments in our study have shown that a proximal versus a distal attentional focus reveals differences in balance performance. Experiment 1 also yielded differences in balance performance between proximal and distal attentional focus in complexity for the hop task. Experiment 2 supported these findings by replicating differences between proximal and distal focus for the CI in COP radius in the hop and step task, respectively. However, classic sway area did not show any difference between foci in one of the tasks. Considering the two different movement effect distances, classic sway variables did not show an effect (besides an interaction effect between both factors) and multiscale entropy CI showed higher values for the more distal condition—associated with more functional balance performance (Busa and van Emmerik, 2016).

Both experiments present an advantageous paradigm, which incorporates classic sway and multiscale entropy sway measures as previous studies assessing attentional focus strategies in balance performance only focused on a linear approach. The presented setting enables more valid comparisons between conditions because context variables are held constant regardless whether subjects focus proximally or distally. Subjects were only instructed to mentally focus on the proximal or distal laser pointer and do not experience any difference in visual input with respect to movement effects between conditions. In general, the instructions only differed by the distinct description of the laser position. Participants knew which laser pointer was switched on and thus created the movement effect via their bodily movements to stay balanced, displayed by the red laser dot on the wall. If both laser pointers would have been activated, they would have intersected in the red dot displayed on the wall. Therefore, it needs to be emphasized that there is only a mental change between conditions but no physical one. However, instructions are chosen to link the proximal condition to a body representation (i.e., laser pointer at forehead) and the distal condition more toward the artificial tool (i.e., laser pointer top of the cone).

According to our findings, the distality of the movement effect on the wall shows an effect on performance in hop and step task. Evidently, the movement effect is critical to voluntary human movement (Wulf and Prinz, 2001). Research has shown that more distal movement effects reveal better performance outcome (McNevin et al., 2003; Bell and Hardy, 2009; McKay and Wulf, 2012). Wulf and Prinz (2001, p. 656) stated that “*on the one hand, the effect should be remote from the body, but on the other hand, the effect should still be so close to producing body action and can still be associated by the learner*.” *As an example*, when using golf tasks in the AF literature with its cues to focus either on club head or more proximal club regions, subjects can visually (even if it is peripheral) focus on both cues in both conditions. In extension to these findings, our studies can disentangle proximal and distal attentional focus effects as the location of AF is out of visual control. Our experiments can therefore differentiate between the distality effects of visual (movement effect) and mental attentional focus cues. The movement effect displayed by the laser dot can be seen as a visual reference point in the environment which has been shown to be crucial for balance tasks. This also supports the findings that the distality of the movement effects influences balance performance with a distinct advantage for the more distal conditions. However, the findings can only be related to single leg dynamic postural control tasks and are not necessarily generalizable (see specificity of attentional focus cues, Raab, 2007 in Wulf et al., 2007, target article).

As presented, the distality of the coordinates of the actual point of focus on an artificial tool showed less of an effect compared to the distality of the visually displayed movement effect. As the differences in the first dimension of distality (point of focus) were correctly expected to be small, statistical differences in the second dimension (movement effect) were surprisingly high. Recent attentional focus literature addressed the phenomenon of external compared to internal focus being superior to performance by considering the importance of the movement effect. Common-coding theory by Prinz (1997) and event coding theory by Hommel et al. (2001) theoretically apply for that notion by postulating that movements need to be planned in terms of their effects (pre-determined outcome) in order to achieve a higher amount of compatibility of planning, action, and perception. In the presented paradigm, participants had visual control on the movement effect via an actual laser pointer as it created the effect. Thus, they build associations between the displayed effect and their own voluntary movements by coupling visually perceived laser movements with sensory feedback of the body. Moreover, on a conceptual basis, associations between the online laser movement on the wall (effect), the laser pointer (effector), and all body movements involving all degrees of freedom in these complex dynamic balance tasks were fostered.

Methodologically, awareness has grown that traditional analysis techniques do not reveal the full information inherent in the signal. So, multivariate alternatives have been considered more frequently. Linear approaches assume that movement (COP sway) is not random but structured and therefore higher sway values would lead to poorer performance. However, these methods do not take any temporal structure of the signal or dynamical postural fluctuations into account. Non-linear dynamic approaches on the other hand address that chaotically driven processes of postural sway across timescales. In these, higher levels of postural sway do not necessarily lead to decreased postural control. By referring to the terminology of complexity, a high amount of random fluctuations within the signal display healthy behavior. Moreover, with respect to attentional processes, it is stated that the more regular the signal becomes the more attentional control is invested (Donker et al., 2007). Busa and van Emmerik (2016) supported this notion by proposing that higher numbers of complexity indicate more autonomous movement control. When referring to common attentional focus theories such as the CAH, a constrained multiscale entropy signal which indicates “worse” balance performance would be associated with a lower CI. Henceforth, lower regularity of the signal as found for the wall 5 m distance can be linked to more functional performance.

The results lead to a consideration about the functionality of complexity. As classic learning approaches associate a decrease in variability of a signal (movement execution) with learning, non-linear models strengthen the importance of variability as being essential to performance in terms of adaptability (Van Emmerik and Van Wegen, 2000). However, the concept of variability can be represented as an inverted U-shaped relationship: a continuous lack of variability can indicate inflexible behavior and an excessive amount of variability creates a highly random and unfocused system which does not foster movement execution (Stergiou et al., 2006). Within boundaries of the base of support, high sway values do not necessarily indicate “worse” performance as it is decisive to analyze the underlying pattern. Vice versa, high sway values cannot always be considered as beneficial to postural control.

Our experiments do not solely rely on the mentioned recent multiscale entropy findings, but the findings are supported by additional movement effect data. Laser data can be seen as primary performance outcome measure as subjects were instructed this way. Even though wall 5 m condition revealed greater laser sway values in the raw data, relatively seen they were smaller than in the 2.5 m condition (applying the intercept theorem). These findings are supported by higher time on target values for the 5 m condition. Subsequently, within the movement effect distality dimension of attentional focus the wall 2.5 m condition showed lower time on target, relatively speaking higher sway values in laser as well as lower values in complexity indices. On the other hand, the wall 5 m condition yielded higher time on target plus higher values in complexity indices.

## Conclusion

As shown in both experiments, the distality of attentional focus cues influences balance performance. Therefore, both experiments can broaden the existing knowledge about the complexity and specificity of attentional focus cues. Also, they underline the relevance of the movement effect to guide motor behavior.

## Data Availability Statement

The datasets generated for this study are available on request to the corresponding author.

## Ethics Statement

The studies involving human participants were reviewed and approved by the Ethics Advisory Committee, Central Washington University, US Ethics Committee Department 7, University of Muenster, Germany. The patients/participants provided their written informed consent to participate in this study.

## Author Contributions

CK contributed to the study design, data acquisition, data analysis, and manuscript production. KR and KZ contributed to the study design, data analysis, and manuscript production. EJ contributed to the data acquisition, data analysis, and manuscript approval.

## Conflict of Interest

The authors declare that the research was conducted in the absence of any commercial or financial relationships that could be construed as a potential conflict of interest.
